# Phenotype, disease severity and pain are major determinants of quality of life in Fabry disease: results from a large multicenter cohort study

**DOI:** 10.1007/s10545-017-0095-6

**Published:** 2017-10-16

**Authors:** Maarten Arends, Simon Körver, Derralynn A. Hughes, Atul Mehta, Carla E. M. Hollak, Marieke Biegstraaten

**Affiliations:** 1 Department of Endocrinology and Metabolism, Academic Medical Center, Meibergdreef 9, 1105 AZ Amsterdam, The Netherlands; 2 Department of Haematology, Royal Free London NHS Foundation Trust, London, UK; 3 Department of Haematology, University College London, London, UK

## Abstract

Quality of life (QoL) is decreased in patients with Fabry disease (FD). To improve QoL, it is important to understand the influence of FD related characteristics, symptoms, and complications. In this retrospective cohort study we explored the effect of pain (measured by the Brief Pain Inventory), phenotype, treatment, and FD-related complications on QoL. QoL data of Fabry patients as assessed by the EuroQol five dimension questionnaire (EQ-5D) from two international centers of excellence were collected. The aim of this study was to evaluate the effect of sex, phenotype, age, different states of disease severity, pain, and ERT on EQ-5D utilities. For 286 adult FD patients (mean age 42.5 years, 40% men, 60% classical phenotype) 2240 EQ-5Ds were available. QoL is decreased in men as well as women with FD, especially in older men with a classical phenotype. At age 50, utility was lower in men with classical FD compared to those with non-classical disease (β = −0.12, 95% CI: -0.23 – 0.01, *p* = 0.037) with further difference in the years thereafter. Cardiovascular complications, stroke or transient ischemic attacks, multiple FD-related complications and pain were also associated with decreased utilities. Overall, no change in utility was seen in patients on ERT over a mean follow-up of 6.1 years. FD leads to a decreased QoL compared to the general population. Disease complications and pain both negatively influence QoL. Adequate assessment and treatment of pain as well as improved strategies to prevent disease complications are needed to improve QoL in the FD population.

**Electronic supplementary material:**

The online version of this article (10.1007/s10545-017-0095-6) contains supplementary material, which is available to authorized users.

## Introduction

Fabry disease (FD; OMIM 301500) is a rare X-linked lysosomal storage disorder with a heterogeneous disease course. The disease is caused by a deficiency of the enzyme α-galactosidase A (enzyme commission no. 3.2.1.22) due to mutations in the GLA-gene. This results in accumulation of globotriaosylceramide (Gb3) and related sphingolipids in cells throughout the body and may cause clinical complications, especially in kidney, heart, and brain. Despite the X-linked inheritance pattern, women are affected as well, and may develop similar symptoms and complications as men (MacDermot et al 2001a; Mehta et al 2004). Both men and women with FD experience a decreased QoL (Street et al 2006; Wang et al 2007; Wilcox et al 2008; Arends et al 2015).

Phenotypically, FD can be divided into classical or non-classical disease. Men with classical FD generally have no residual enzyme activity and often exhibit Fabry-specific symptoms including neuropathic pain, cornea verticillata, and angiokeratoma. Men with non-classical FD and women with either classical or non-classical FD have residual enzyme activity, usually resulting in a milder disease course. Older studies showed severely decreased QoL, predominantly in men who nowadays most likely would be considered to have classical FD (Gold et al 2002; Miners et al 2002). Also in more recent studies a distinction in phenotypes has not been made. In other words, the effect of phenotype on QoL has yet to be elucidated.

Part of the decreased QoL in patients with FD seems to be associated with the neuropathic pain often seen in men with classical FD (Gold et al 2002). Episodes of severe, debilitating burning pains can be alternated with chronic pain, mostly in hands and feet. Moreover, the presence of gastro-intestinal (GI) symptoms, including GI-pain, is also associated with lower QoL (Hoffmann et al 2007). Several studies reported a positive effect of enzyme replacement therapy (ERT) on pain (Schiffmann et al 2001; Hoffmann et al 2005; Watt et al 2010). In contrast, no clear effect of ERT on QoL was established in a recent systematic review from our group (Arends et al 2015). Besides pain, complications linked to FD such as end stage renal disease (ESRD), cardiomyopathy, and stroke have been associated with decreased QoL (Gold et al 2002; Miners et al 2002; Wagner et al 2014). For the purpose of a cost-effectiveness analysis, Rombach et al created mutually exclusive disease states to simulate the disease course of FD (Rombach et al 2013a). Lower QoL was found in patients in a more severe disease state. However, the sample size necessitated grouping of different complications in one single group, so the effect of individual cerebral, renal or cardiac complications on QoL remained unknown. Better understanding of QoL in different disease states and improved understanding of the influence of specific symptoms and complications on QoL may facilitate targeted treatment, and thereby improve the well-being of Fabry patients. With this study we aim to gain insight into the influence of sex, phenotype, age, disease severity, and ERT on QoL.

## Methods

### Study design

Using local databases containing prospectively collected data as well as medical records, demographic, clinical, and laboratory data of all FD patients from two centers of excellence (Academic Medical Center (AMC), The Netherlands; and Royal Free London NHS Foundation Trust (RFH), United Kingdom) were merged into one database. This cohort represents part of a larger study (Arends et al 2016) with available EuroQol five dimension questionnaire (EQ-5D) data. Baseline was defined as the date of the first EQ-5D measurement, except for the evaluation of the influence of ERT on the QoL where the start date of ERT was used as baseline.

According to Dutch law, and after review of the AMC ethics committee, no approval of the study protocol was needed because of the observational nature of the study. All data were obtained from medical records. Patient records were anonymized and de-identified prior to analysis. All patients have provided consent for the use of their medical data and samples in accordance with local ethics requirements.

### Study participants

Adult patients (≥18 years) with a definite FD diagnosis according to previously developed criteria (Smid et al 2014) of whom sufficient data for phenotypical classification and one or more EQ-5D measurements were available, were included. They were categorized as classical or non-classical on the basis of enzyme activity and the presence or absence of characteristic FD symptoms (Fabry neuropathic pain, clustered angiokeratoma and/or cornea verticillata (van der Tol et al 2014)). A detailed description of the classification method has been published earlier (Supplement A, (Arends et al 2016)).

### EQ-5D

The EQ-5D is a QoL questionnaire that covers five different QoL domains: mobility, self-care, anxiety/depression, usual activities, and pain/discomfort (EuroQol-Group 1990). Respondents are asked to choose per domain which one of the following three options describes their situation best: no problems, some/moderate problems or extreme problems (Rabin and de Charro 2001). EQ-5D data can be presented as a health profile which shows the frequency of reported problems for each level for each dimension (Rabin and de Charro 2001). Also, a utility for the health status can be calculated by combining the responses on all five domains. A utility of 1 means perfect health and a score of 0 represents death. Negative scores can also be obtained representing health states that are considered worse than death. Utilities differ per country. For our study we used the Dutch and UK weighing for Dutch and English patients, respectively (Dolan 1997; Lamers et al 2005).

### Pain assessment

The AMC and the RHF both used the Brief Pain Inventory (BPI) to assess the presence and severity of pain and its influence on daily life. All BPI scores closest to the utility with a maximum window of ±3 months were used. The BPI assesses pain at its worst, average pain, and pain interference with life. The interference score measures the influence of pain on general activity, walking, work, mood, enjoyment of life, relations and sleep. It is the mean of at least four of these items. Worst pain, average pain, and the interference score are graded from 0 (pain is absent) to 10 (worst possible pain) (Cleeland and Ryan 1994).

### Disease severity

To evaluate the effect of symptoms, organ involvement, and complications in FD, patients were classified in ten mutually exclusive disease states with increasing severity (Rombach et al 2013a) (Table 1). According to strict criteria, patients can transition from one state to another in case of disease progression.

**Table 1:** Description of disease states

Disease state*	Description
*Asymptomatic*	
No organ involvement	No left ventricular hypertrophy, kidney disease, white matter lesions or complications
*Symptoms*	
Neuropathic pain	A history of Fabry neuropathic pain in the extremities provoked by heat, fever or exercise (also referred to as acroparesthesia)
Organ involvement	Left ventricular hypertrophy, chronic kidney disease stages 2–4, albuminuria/proteinuria or white matter lesions
*Single complication*	
End stage renal disease	Chronic kidney disease stage 5 (eGFR < 15 ml/min/1.73m^2^), dialysis or kidney transplant
Cardiac complication(s)	Atrial fibrillation, any other rhythm disturbance needing hospitalization, pacemaker or implantable cardiac defibrillator (ICD) implantation, cardiac congestion for which hospital admittance was needed, myocardial infarction, percutaneous coronary intervention or coronary artery bypass graft
Cerebrovascular accident	Transient ischemic attack (TIA) or stroke, as diagnosed by a neurologist
*Multiple complications* ^#^	
End stage renal disease and cardiac complication(s)	
End stage renal disease and cerebrovascular accident	
Cardiac complication(s) and cerebrovascular accident	
End stage renal disease and cardiac complication(s) and cerebrovascular accident	

*Typically, patients progress from the asymptomatic state or neuropathic pain state to the symptoms state; from the symptoms state to a single complication state; from a single complication state to a double complication state, and from a double complication state to the triple complication state

^#^Since the number of patients in the disease states representing more than one complication was low, one combined ‘multiple complications’ disease state was made

### Clinical and laboratory measurements

Renal function was evaluated by the estimated glomerular filtration rate (eGFR) using the CKD-EPI formula (Kidney Disease: Improving Global Outcomes (KDIGO) CKD Work Group 2013) and proteinuria. Cardiac involvement was assessed by echocardiography. Left ventricular mass (LVM) was calculated using the Devereux formula and was corrected for height (m^2.7^) (Lang et al 2005). Left ventricular hypertrophy was defined as LVM ≥49 and ≥45 g/m^2.7^ in men and women, respectively (Lang et al 2005). The presence of white matter lesions (WMLs)/ischemic lesions was investigated by cerebral MRI. Plasma lysoGb3 levels were measured with tandem mass spectrometry with glycine labeled (RFH and AMC after August 2015) or isotope labeled lysoGb3 (AMC before August 2015) as an internal standard (Gold et al 2013; Arends et al 2016).

### Statistical methods

R (version 3.1.5) and SPSS for Windows, version 22.0 (SPSS Inc. Chicago, Illinois, USA) were used. First, utilities per sex and phenotype were calculated, followed by a second order polynomial regression mixed effect model with a random intercept and slope to evaluate the effect of age on utilities, stratified for sex and phenotype. To evaluate the relation between BPI score and utility, the polynomial mixed effect model was extended by including BPI scores as covariate.

Second, the effect of ERT on utilities was investigated with a linear mixed model including time on ERT and age at ERT initiation as time-dependent covariates and a mixed model of the difference between the utility at baseline and follow up measurements. Since QoL is known to fluctuate over time, patients were only included in this analysis if they completed an EQ-5D within 3 months before the start of ERT. Finally, utilities per disease state for the combined cohort of men and women with classical and non-classical disease were modeled. To account for the fact that one patient may have filled in more than one EQ-5D per disease state, a linear mixed effect model with the disease state as covariate and a random intercept was used to evaluate the utility per disease state. Patient numbers were too small to include sex and phenotype. In order to analyze the effect of eGFR, LVM, and WML on QoL within the “organ involvement” disease state, univariate, and multivariate analyses were performed within this group. Data are presented as mean ± standard deviation (SD) or median and range dependent on the distribution of data. Where appropriate, 95% confidence intervals (95% CI) are given. *P*-values <0.05 were considered statistically significant.

## Results

The merged database contained data on 439 patients from the AMC and the RFH, of whom 27 patients did not fulfill the criteria for a definite diagnosis, 12 had insufficient baseline data for assessment of disease severity and no follow-up data, and 114 patients did not complete one or more EQ-5D measurements. Two-hundred-eighty-six FD patients (117 patients from the AMC and 169 patients from the RFH) with a mean age: 42.5 ± 12.5 years completed 2240 EQ-5Ds. Each patient completed on average 7.8 ± 4.5 EQ-5Ds during a mean follow-up period of 5.4 ± 3.2 years. Classically affected patients completed more EQ-5Ds compared to non-classically affected patients. Table 2 shows the baseline characteristics at the time of completion of the first EQ-5D.

**Table 2:** Characteristics of all patients at first EQ-5D measurement

	All	Men		Women	
		Classical	Non-classical	Classical	Non-classical
Patients, n (%)	286	76 (26.6)	38 (13.3)	96 (33.6)	76 (26.6)
Age in years, mean (±SD)	42.5 (±15.5)	37.4 (±12.5)	54.2 (±15.4)	44.0 (±15.5)	40.7 (±15.2)
Age first visit, mean (±SD)	40 (±16.0)	34.2 (±12.5)	52.8 (±15.8)	42.6 (±15.6)	37.8 (±15.5)
History of ERT, n (%)	125 (43.7)	57 (75.0)	13 (34.2)	41 (42.7)	14 (18.4)
Currently on ERT, n (%)	117 (40.9)	52 (68.4)	13 (34.2)	39 (40.6)	13 (17.1)
Time on ERT in years, mean (±SD)	2.97 (±2.38)	3.69 (±2.61)	1.94 (±2.03)	2.73 (±2.05)	1.87 (±1.89)
Events before first EQ-5D					
Any event, n (%)	50 (17.5)	16 (21.1)	14 (36.8)	16 (16.7)	4 (5.3)
Cardiac event, n (%)	33 (11.5)	8 (10.5)	12 (31.6)	9 (9.4)	4 (5.3)
Renal event, n (%)	8 (2.8)	5 (6.6)	2 (5.3)	1 (1.0)	0 (0)
Cerebral event, n (%)	20 (7.0)	8 (10.5)	3 (7.9)	9 (9.4)	0 (0)
WML, n (%)	112 (39.2)	34 (44.7)	11 (28.9)	41 (42.7)	26 (34.2)
eGFR in ml/min/1.73m^2^, mean (±SD)	93.8 (±25.9)	99.0 (±30.3)	80.6 (±26.3)	96.7 (±21.6)	91.5 (±23.7)
eGFR <60 ml/min/1.73m^2^, n (%)	29 (10.3)	10/76 (13.2)	6/38 (15.8)	5/94 (5.3)	8/73 (11.0)
LVM in gr/m^2.7^, median (range)	42.0 (16.2–139.9)	48.3 (23.6–110.4)	54.2 (16.2–99.9)	40.8 (18.2–139.9)	34.5 (17.1–96.2)
LVM > upper ref. limit, n (%)	101 (37.4)	33/74 (44.6)	20/35 (57.1)	32/92 (34.8)	16/69 (23.2)
LysoGb3* in nmol/L, median (range)	7.5 (0.4–150.3)	105.5 (31.8–150.3)	6.0 (1.2–22.4)	7.6 (0.7–27.2)	2.0 (0.4–15.4)
BPI average pain*, median (range)	2 (0–8)	2 (0–8)	0 (0–7)	3 (0–8)	3 (0–8)
BPI worst pain*, median (range)	3 (0–10)	3 (0–10)	0 (0–9)	3 (0–9)	3 (0–10)
BPI average interference*, median (range)	0.6 (0–9.9)	0.5 (0.0–8.4)	0.1 (0.0–9.9)	0.5 (0.0–9.3)	1.1 (0.0–9.7)
EQ-5Ds^#^, n	2240	668	286	771	515
EQ-5Ds per patient^#^, mean (±SD)	7.8 (±4.5)	8.8 (±4.8)	7.5 (±4.1)	8.2 (±4.5)	6.8 (±4.4)
Follow-up time^#^, mean (±SD)	5.38 (±3.15)	5.73 (±3.46)	4.71 (±2.86)	5.56 (±2.98)	5.13 (±3.15)

Events represent the number of patients with one or more events before first EQ-5D. Events were defined as described at end stage renal disease, cardiac complications, and cerebrovascular accident similar to the definition of the disease states (Table 1)

*ERT*, enzyme replacement therapy; *WML*, white matter lesions on MRI; *eGFR*, estimated glomerular filtration rate; *LVM*, left ventricular mass index on echocardiography; *BPI*, Brief Pain Inventory

Upper reference limit LVM: ♂ = 51 / ♀ = 48. Normal range lysoGb3 = 0.3–0.6 nmol/L. LysoGb3 represents values before start of ERT.

*Values missing: LysoGb3 32%, BPI average pain 16%, BPI worst pain 15%, BPI average interference 12%

^#^Values acquired during follow-up

### Health profile at baseline per phenotype and per disease state

The health profile of the first completed EQ-5D (Table 3) shows that 35.5% of all men with classical FD reported some/moderate problems of mobility with one patient reporting extreme problems. Lower percentages (21.9% to 28.9%) of women and men with non-classical disease reported some/moderate mobility problems. Self-care was relatively preserved in all subgroups of patients, while 35.3% experienced some/moderate problems with their usual activities with percentages ranging from 25.0% in women with non-classical FD to 42.1% in men with classical disease. Twenty-one patients (7.3%) experienced extreme pain at baseline and almost two-thirds of the men with classical FD experienced at least some/moderate pain. Some/moderate anxiety or depressive symptoms were noted in about one third of men with classical FD and women with classical and non-classical FD. The health profiles of the first EQ-5D per disease state (Supplemental Table B) indicated that a large proportion of patients in the ‘neuropathic pain’ disease state reported problems with their usual activities and anxiety/depression domain, and some/moderate to extreme problems in the pain/discomfort domain. Since the number of patients in the disease states representing more than one complication was low, one combined ‘multiple complications’ disease state was made, representing patients with complications in at least two organs (kidney, heart and/or brain). Patients in the ‘single’ and ‘multiple complication’ disease states reported problems across all domains.

**Table 3:** Health profile and utility of the first EQ-5D measurement stratified for sex and phenotype

	All	Men		Women	
		Classical	Non-classical	Classical	Non-classical
Mobility	Number of patients (%)				
1*	207 (72.4)	48 (63.2)	27 (71.1)	75 (78.1)	57 (75.0)
2	78 (27.3)	27 (35.5)	11 (28.9)	21 (21.9)	19 (25.0)
3	1 (0.3)	1 (1.3)	0 (0.0)	0 (0.0)	0 (0.0)
Self-care	Number of patients (%)				
1	261 (91.3)	64 (84.2)	34 (89.5)	93 (96.9)	70 (92.1)
2	22 (7.7)	10 (13.2)	4 (10.5)	3 (3.1)	5 (6.6)
3	3 (1.0)	2 (2.6)	0 (0.0)	0 (0.0)	1 (1.3)
Usual activities	Number of patients (%)				
1	173 (60.5)	40 (52.6)	25 (65.8)	57 (59.4)	51 (67.1)
2	101 (35.3)	32 (42.1)	12 (31.6)	38 (39.4)	19 (25.0)
3	12 (4.2)	4 (5.3)	1 (2.6)	1 (1.0)	6 (7.9)
Pain/discomfort	Number of patients (%)				
1	138 (48.3)	29 (38.2)	22 (57.9)	47 (49.0)	40 (52.6)
2	127 (44.4)	43 (56.6)	15 (39.5)	40 (41.7)	29 (38.2)
3	21 (7.3)	4 (5.3)	1 (2.6)	9 (9.4)	7 (9.2)
Anxiety/depression	Number of patients (%)				
1	191 (66.8)	51 (67.1)	27 (71.1)	65 (67.7)	48 (63.2)
2	90 (31.5)	25 (32.9)	9 (23.7)	31 (32.3)	25 (32.9)
3	5 (1.7)	0 (0.0)	2 (5.3)	0 (0.0)	3 (3.9)
Utility^#^, mean (±SD)	0.77 (±0.26)	0.75 (±0.25)	0.81 (±0.27)	0.79 (±0.23)	0.76 (±0.30)

*1 = no problems, 2 = some/moderate problems, 3 = extreme problems

**Fig. 1:**
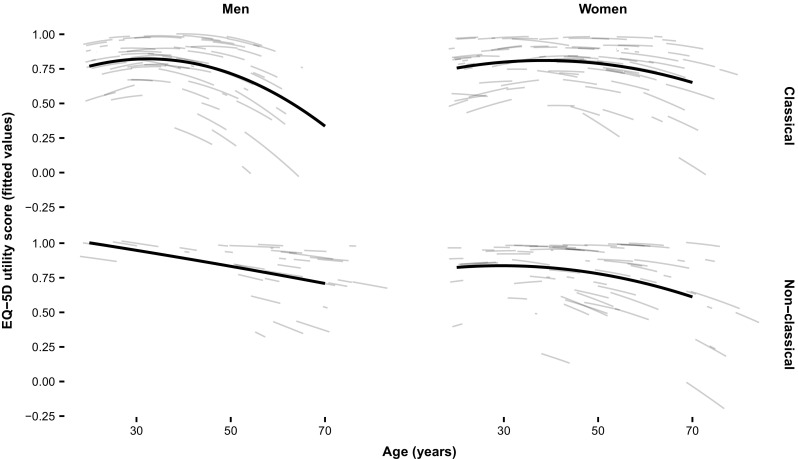
Relation between EQ-5D and age. Polynomial mixed effect model of EQ-5D in relation to age, stratified for sex and phenotype. Large lines represent fitted values at group level, the smaller lines represent the fitted values at individual patient level

^*#*^ Please note that a baseline utility is presented in contrast to Fig. 1 which presents longitudinal utilities

### Relation between phenotype, age, and utilities

A decrease in utilities with age was seen in all subgroups (Fig. 1), although the extent of this relation differed between the subgroups. The mixed model revealed that the utility of a 50-year-old man with classical FD is on average 0.12 points lower (95% CI: -0.23 – 0.01, *p* = 0.037) compared to a man with non-classical FD of the same age. At age 60 this mean difference has increased to 0.21 points (95% CI: -0.36 – -0.07, *p* = 0.004). This illustrates the progressive worsening of utilities in men with classical FD with increasing age. In women and men with non-classical FD the decline in utilities with age was less strong (Fig. 1).

### Relation between BPI scores and utilities

In total 1559 BPIs of 276 patients were matched to an EQ-5D. Of the patients that completed BPIs 45.3% were men, 61.2% had the classical phenotype, and the mean age was 44.8 ± 14.9 years. Utilities decreased with higher BPI scores: with every one point higher BPI average pain score, the utility decreased on average by 0.045 points (β = −0.045, 95% CI: -0.049 – -0.040, *p* < 0.001). Similarly, an increase in BPI worst pain score (β = −0.035, 95% CI: -0.039 – -0.031, *p* < 0.001) or BPI interference score (β = −0.058, 95% CI: -0.063 – -0.053, *p* < 0.001) resulted in lower utilities. A sensitivity analysis without the pain/discomfort domain in the calculation of the utilities did not change the results.

### The effect of ERT on utilities

For the evaluation of the effect of ERT on utilities, 61 patients were analyzed who completed at least one EQ-5D before the initiation of ERT. The mean age of patients at ERT initiation was 44.2 ± 15.5 years, and the mean follow-up time after initiation of ERT was 6.1 ± 2.5 years. The median utility score before ERT was 0.796 (−0.166–1.000). Utility remained unchanged after start of treatment (β = −0.004, 95% CI: -0.066 – 0.058, *p* = 0.89). Furthermore, there was no relation between change in utility, time on ERT (β = −0.005, 95% CI: -0.016 – 0.006, *p* = 0.40) and age at ERT initiation (β = −0.002, 95% CI: -0.006 – 0.001). In a subgroup analysis, we found that utilities increased after initiation of ERT in the 13 men with classical FD (β = 0.17, 95% CI: 0.0 6– 0.28, *p* = 0.003). This was primarily attributable to three patients with a very low utility before the start of ERT due to extreme pain/discomfort which improved substantially after the start of treatment. One of them started taking carbamazepine for his neuropathic pain during the same period, leading to a substantial decrease in neuropathic pain in the months thereafter. Without these three patients no change in utility was observed (β = 0.04, 95% CI: -0.07 – 0.14, *p* = 0.50). Additional subgroup analyses revealed that no annualized change in utility was observed in women with classical or non-classical disease, while in men with non-classical FD a 0.027 point decline per year on ERT (95% CI: -0.053 – 0.001, *p* = 0.04) was found.

### Relation between disease severity and utilities

Table 4 shows the mean utility per disease state for men and women with classical and non-classical FD combined. Within the “organ involvement” disease state we found no relation between eGFR, LVMI, and WML on the one hand and utilities on the other hand. Patients included in the advanced disease states, and thus with more severe disease, were older and more often men with classical FD. Compared to the ‘no organ involvement’ disease state, the utilities were significantly lower in the ‘cardiac complication(s)’, ‘cerebrovascular accident’, and the ‘multiple complications’ disease states but not the ‘end stage renal disease’ disease state. The utility of the latter was based on a low number of patients (*n* = 7) who showed divergent utility scores. The lowest utility was found in the ‘multiple complications’ disease state (β = 0.530, 95% CI: 0.42–0.63). The ‘neuropathic pain’ disease state also showed a trend toward lower utility compared to the ‘no organ involvement’ disease state (*p* = 0.053).

**Table 4:** Utility per disease state

Disease state	No organ involvement	Neuropathic pain	Organ involvement	End stage renal disease	Cerebrovascular accident	Cardiac complication(s)	Multiple complications
Patients*, n	31	21	221	7	16	45	18
EQ-5Ds, n	103	71	1521	56	100	290	99
Health utility, mean (95% CI)	0.851 (0.77–0.93)	0.725 (0.63–0.82)	0.783 (0.75–0.81)	0.828 (0.67–0.99)	0.705 (0.60–0.81)	0.732 (0.67–0.80)	0.530 (0.42–0.64)
*P*-value^#^	–	0.053	0.123	0.796	**0.037**	**0.026**	**<0.001**
Woman, n (%)	26 (83.9)	15 (71.4)	138 (62.4)	1 (14.3)	8 (50.0)	20 (44.4)	6 (33.3)
Classical phenotype, n (%)	5 (16.1)	19 (90.5)	136 (61.5)	6 (85.7)	15 (93.8)	21 (46.7)	14 (77.8)
Age in years, mean (±SD)	32.0 (±10.1)	26.5 (±8.6)	41.0 (±14.1)	45.8 (±12.9)	49.3 (±10.1)	59.2 (±11.0)	60.5 (±8.4)
History of ERT, n (%)	3 (9.7)	2 (9.5)	107 (48.4)	5 (71.4)	13 (81.2)	32 (71.1)	14 (77.8)
Now ERT, n (%)	3 (9.7)	2 (9.5)	97 (43.9)	5 (71.4)	13 (81.2)	31 (68.9)	13 (72.2)
Time on ERT in years, mean (±SD)	1.40 (±0.49)	0.98 (±1.31)	2.72 (±2.14)	3.06 (±1.72)	4.67 (±4.08)	4.91 (±4.04)	6.14 (±2.81)

Numbers in bold are considered statistically significant (*p*<0.05)

*ERT*, enzyme replacement therapy

* Patients may have more than one EQ-5D per disease state and may contribute to more than one disease state. ^#^ *P*-values were calculated with ‘no organ involvement’ as reference group

## Discussion

This study shows that QoL in patients with FD is related to phenotype, age, pain, and disease severity. Obviously, these features are related to each other; classically affected patients of older age will have more severe disease with a higher chance of developing FD-related complications and thus a decreased QoL. Additional analyses to study the independent effects of these features on QoL were not feasible due to limited patient numbers and the expectation of high multicollinearity.

The mean utility of FD patients in the present study ranged from 0.75 in men with classical FD to 0.81 in men with non-classical disease, and QoL decreased with advancing age. Comparison of the health profile of these patients to the health profile of the general population in the UK and the Netherlands supports a higher prevalence of impaired QoL in FD (Supplemental Table C) (Kind et al 1998; Lamers et al 2006). In line with the current results, a recent study in a mixed cohort of treated and untreated men and women with FD showed a mean utility of 0.79 (Żuraw et al 2011).

In contrast, a cohort study from the pre-ERT era in which 38 men of similar age and with presumed classical disease were included, showed a substantially lower utility of 0.56 (Miners et al 2002). These patients had more often suffered from one or more complications (Miners et al 2002). Therefore, a possible explanation for the difference in utilities is that in our cohort ERT delayed the occurrence of complications and therefore delayed the decrease in QoL caused by complications (Rombach et al 2013b). Indeed, in a recent study from our group on the natural course of FD stratified by sex and phenotype, men with classical disease were shown to have the highest risk of developing complications (Arends et al 2016). The median age at first complication was approximately 50 years, which corresponds with the age after which the decrease in QoL accelerates in these men in the present study. The fact that the utility in the pre-ERT cohort resembles the utility in our ‘multiple complications’ disease state further supports this. It is unlikely that QoL scores in the investigated FD populations are influenced by regional differences, since the QoL scores in the general population in the UK and the Netherlands are comparable (Supplemental Table C). However, other factors may have contributed to the higher QoL in the present study compared to the pre-ERT cohort, such as pain management. Indeed, a detailed look at the health profiles of the different subgroups of patients indicates that the prevalence of extreme pain seems to have decreased when compared to older studies (Supplemental Table C) (Miners et al 2002; Hoffmann et al 2005). However, pain is still present in around half of the patients and associated with lower QoL in FD, as also established in a Fabry Outcome Survey study (Hoffmann et al 2005). Moreover, chronic pain is related to decreased QoL in all domains in the general population (O’Brien and Breivik 2012). Therefore, QoL is expected to increase if pain control is improved. An often mentioned cause of pain in patients with FD is neuropathic pain in the extremities (also called acroparesthesia), which is associated with decreased QoL (Gold et al 2002). However, in this study, men with non-classical FD and women, who are known to have a low prevalence of Fabry-related neuropathic pain, also frequently reported pain. Indeed, a previous study has shown that other types of pain (e.g., musculoskeletal pain or GI-pain) may play an important role in the life of FD patients (Uceyler et al 2014). As a consequence, it is recommended to assess individual causes of pain and manage it accordingly (Uceyler et al 2014; Politei et al 2016; Schuller et al 2016).

The reported percentage of extreme anxiety/depression in the health profile has also decreased compared to older studies (Miners et al 2002; Hoffmann et al 2005). The availability of treatment in itself can provide hope or relief of complaints and might reduce anxiety/depressive complaints (Miller et al 2009). On the other hand, in other metabolic diseases it has been speculated that biweekly infusions are burdensome, especially in the hospital setting, thereby potentially affecting QoL (Angelini 2015; Gungor et al 2016). In our study, no effect of ERT was seen on the percentage of patients with anxiety/depression.

Previous studies have not been able to unequivocally determine the effect of ERT on QoL (Arends et al 2015). The current study showed that QoL did not change over 6 years of follow-up in patients receiving ERT. However, there are individual differences in the course of QoL with some patients deteriorating while others improve. Of interest to this end is the observation that three out of 13 men with classical FD had higher utilities after start of ERT, especially in the pain and activity domain. However, it is difficult to attribute the improvement in QoL to the start of ERT, because pain may subside spontaneously (MacDermot et al 2001b; Biegstraaten et al 2012). Moreover, we could not correct for concomitant analgesic or antidepressant treatment that might improve or stabilize QoL.

In patients with deteriorating QoL, ERT may have been started too late. Indeed, it has been previously shown that ERT is of limited benefit in patients with advanced organ involvement and complications (Rombach et al 2013b). In the present study complications are clearly associated with a decreased QoL: cerebrovascular accidents, cardiovascular complications, and multiple complications resulted in a decrease in utilities of 0.15, 0.12, and 0.32 respectively, all exceeding the minimally clinically important difference of 0.074 (Walters and Brazier 2005).

Our study has several limitations. Firstly, the EQ-5D offers three answer options per domain, limiting the detection of small changes in health (Herdman et al 2011). Secondly, questionnaire data were gathered during clinical visits, in some patients for up to 15 years in a row. Habituation to repeatedly filling out a questionnaire as well as coping might have influenced the questionnaire accuracy. Thirdly, the lack of a control group hampered the interpretation of the effect of ERT on QoL. Finally, since the QoL data were gathered in an uncontrolled, real-life environment, they were more prone to be influenced by known and unknown confounders, such as presence of concomitant diseases and the use of pain medication. On the other hand, real-life data provides an opportunity to assess QoL in actual practice conditions. Despite these shortcomings an insight has been gained into QoL in FD and its determinants, which can be used to improve the care for these patients.

## Conclusion

In conclusion, QoL is decreased in men as well as women with FD compared to the general population, especially in older men with a classical phenotype. QoL is lower in patients with FD-related complications and ERT does not seem to have a major impact on QoL. This necessitates the improvement of treatment and preventive strategies. Pain also has a severe impact on QoL. It is prevalent in both sexes and phenotypes and comprises more than neuropathic pain alone. Pain assessment should be an important part of routine follow-up and treatment should be standardized and evaluated accordingly.

## Electronic supplementary material

ESM 1 (PDF 70 kb)

ESM 2 (PDF 99 kb)

ESM 3 (PDF 154 kb)

## References

[CR1] Angelini C (2015). Spectrum of metabolic myopathies. Biochim Biophys Acta.

[CR2] Arends M, Hollak CE, Biegstraaten M (2015). Quality of life in patients with Fabry disease: a systematic review of the literature. Orphanet J Rare Dis.

[CR3] Arends Maarten, Wanner Christoph, Hughes Derralynn, Mehta Atul, Oder Daniel, Watkinson Oliver T., Elliott Perry M., Linthorst Gabor E., Wijburg Frits A., Biegstraaten Marieke, Hollak Carla E. (2016). Characterization of Classical and Nonclassical Fabry Disease: A Multicenter Study. Journal of the American Society of Nephrology.

[CR4] Biegstraaten M, Hollak CE, Bakkers M, Faber CG, van Schaik IN (2012). Small fiber neuropathy in Fabry disease. Mol Genet Metab.

[CR5] Cleeland CS, Ryan KM (1994). Pain assessment: global use of the Brief Pain Inventory. Ann Acad Med Singap.

[CR6] Dolan P (1997). Modeling valuations for EuroQol health states. Med Care.

[CR7] EuroQol-Group (1990). EuroQol-a new facility for the measurement of health-related quality of life. Health Policy.

[CR8] Gold H, Mirzaian M, Dekker N (2013). Quantification of globotriaosylsphingosine in plasma and urine of fabry patients by stable isotope ultraperformance liquid chromatography-tandem mass spectrometry. Clin Chem.

[CR9] Gold KF, Pastores GM, Botteman MF (2002). Quality of life of patients with Fabry disease. Qual Life Res.

[CR10] Gungor D, Kruijshaar ME, Plug I (2016). Quality of life and participation in daily life of adults with Pompe disease receiving enzyme replacement therapy: 10 years of international follow-up. J Inherit Metab Dis.

[CR11] Herdman M, Gudex C, Lloyd A (2011). Development and preliminary testing of the new five-level version of EQ-5D (EQ-5D-5L). Qual Life Res.

[CR12] Hoffmann B, Garcia de Lorenzo A, Mehta A, Beck M, Widmer U, Ricci R (2005). Effects of enzyme replacement therapy on pain and health related quality of life in patients with Fabry disease: data from FOS (Fabry Outcome Survey). J Med Genet.

[CR13] Hoffmann B, Schwarz M, Mehta A, Keshav S (2007). Gastrointestinal symptoms in 342 patients with Fabry disease: prevalence and response to enzyme replacement therapy. Clin Gastroenterol Hepatol.

[CR14] Kidney Disease: Improving Global Outcomes (KDIGO) CKD Work Group (2013) KDIGO 2012 Clinical Practice Guideline for the Evaluation and Management of Chronic Kidney Disease. 3:1–150

[CR15] Kind P, Dolan P, Gudex C, Williams A (1998). Variations in population health status: results from a United Kingdom national questionnaire survey. BMJ.

[CR16] Lamers LM, McDonnell J, Stalmeier PF, Krabbe PF, Busschbach JJ (2006). The Dutch tariff: results and arguments for an effective design for national EQ-5D valuation studies. Health Econ.

[CR17] Lamers LM, Stalmeier PF, McDonnell J, Krabbe PF, van Busschbach JJ (2005). Measuring the quality of life in economic evaluations: the Dutch EQ-5D tariff. Ned Tijdschr Geneeskd.

[CR18] Lang RM, Bierig M, Devereux RB (2005). Recommendations for chamber quantification: a report from the American Society of Echocardiography's Guidelines and Standards Committee and the Chamber Quantification Writing Group, developed in conjunction with the European Association of Echocardiography, a branch of the European Society of Cardiology. J Am Soc Echocardiogr.

[CR19] MacDermot KD, Holmes A, Miners AH (2001). Anderson-Fabry disease: clinical manifestations and impact of disease in a cohort of 60 obligate carrier females. J Med Genet.

[CR20] MacDermot KD, Holmes A, Miners AH (2001). Anderson-Fabry disease: clinical manifestations and impact of disease in a cohort of 98 hemizygous males. J Med Genet.

[CR21] Mehta A, Ricci R, Widmer U (2004). Fabry disease defined: baseline clinical manifestations of 366 patients in the Fabry Outcome Survey. Eur J Clin Investig.

[CR22] Miller FG, Colloca L, Kaptchuk TJ (2009). The placebo effect: illness and interpersonal healing. Perspect Biol Med.

[CR23] Miners AH, Holmes A, Sherr L, Jenkinson C, MacDermot KD (2002) Assessment of health-related quality-of-life in males with Anderson Fabry disease before therapeutic intervention. Qual Life Res 11:127–133 10.1023/a:1015009210639 12018736

[CR24] O’Brien Tony, Breivik Harald (2012). The impact of chronic pain—European patients’ perspective over 12 months. Scandinavian Journal of Pain.

[CR25] Politei Juan M., Bouhassira Didier, Germain Dominique P., Goizet Cyril, Guerrero-Sola Antonio, Hilz Max J., Hutton Elspeth J., Karaa Amel, Liguori Rocco, Üçeyler Nurcan, Zeltzer Lonnie K., Burlina Alessandro (2016). Pain in Fabry Disease: Practical Recommendations for Diagnosis and Treatment. CNS Neuroscience & Therapeutics.

[CR26] Rabin R, de Charro F (2001). EQ-5D: a measure of health status from the EuroQol Group. Ann Med.

[CR27] Rombach SM, Hollak CE, Linthorst GE, Dijkgraaf MG (2013). Cost-effectiveness of enzyme replacement therapy for Fabry disease. Orphanet J Rare Dis.

[CR28] Rombach SM, Smid BE, Bouwman MG, Linthorst GE, Dijkgraaf MG, Hollak CE (2013). Long term enzyme replacement therapy for Fabry disease: effectiveness on kidney, heart and brain. Orphanet J Rare Dis.

[CR29] Schiffmann Raphael, Kopp Jeffrey B., Austin III Howard A., Sabnis Sharda, Moore David F., Weibel Thais, Balow James E., Brady Roscoe O. (2001). Enzyme Replacement Therapy in Fabry Disease. JAMA.

[CR30] Schuller Y, Linthorst GE, Hollak CEM, Van Schaik IN, Biegstraaten M (2016) Pain management strategies for neuropathic pain in Fabry disease—a systematic review. BMC Neurol 16:16–25 10.1186/s12883-016-0549-8 PMC4766720 26911544

[CR31] Smid B.E., van der Tol L., Cecchi F., Elliott P.M., Hughes D.A., Linthorst G.E., Timmermans J., Weidemann F., West M.L., Biegstraaten M., Lekanne Deprez R.H., Florquin S., Postema P.G., Tomberli B., van der Wal A.C., van den Bergh Weerman M.A., Hollak C.E. (2014). Uncertain diagnosis of Fabry disease: Consensus recommendation on diagnosis in adults with left ventricular hypertrophy and genetic variants of unknown significance. International Journal of Cardiology.

[CR32] Street NJ, Yi MS, Bailey LA, Hopkin RJ (2006). Comparison of health-related quality of life between heterozygous women with Fabry disease, a healthy control population, and patients with other chronic disease. Genet Med.

[CR33] Uceyler N, Ganendiran S, Kramer D, Sommer C (2014). Characterization of pain in fabry disease. Clin J Pain.

[CR34] van der Tol L, Cassiman D, Houge G (2014). Uncertain diagnosis of fabry disease in patients with neuropathic pain, angiokeratoma or cornea verticillata: consensus on the approach to diagnosis and follow-up. JIMD Rep.

[CR35] Wagner M, Krämer J, Blohm E (2014). Kidney function as an underestimated factor for reduced health related quality of life in patients with Fabry disease. BMC Nephrol.

[CR36] Walters SJ, Brazier JE (2005). Comparison of the minimally important difference for two health state utility measures: EQ-5D and SF-6D. Qual Life Res.

[CR37] Wang RY, Lelis A, Mirocha J, Wilcox WR (2007). Heterozygous Fabry women are not just carriers, but have a significant burden of disease and impaired quality of life. Genet Med.

[CR38] Watt T, Burlina AP, Cazzorla C (2010). Agalsidase beta treatment is associated with improved quality of life in patients with Fabry disease: findings from the Fabry Registry. Genet Med.

[CR39] Wilcox WR, Oliveira JP, Hopkin RJ (2008). Females with Fabry disease frequently have major organ involvement: lessons from the Fabry Registry. Mol Genet Metab.

[CR40] Żuraw W, Golicki D, Jurecka A, Tylki-Szymańska A (2011) Quality of life among polish Fabry patients—a cross-sectional study quality of life among polish Fabry patients. Cent Eur J Med 6:741–749

