# Supplementary material for: Phenotype, disease severity and pain are major determinants of quality of life in Fabry disease: results from a large multicenter cohort study
Source: J Inherit Metab Dis. 2017 Oct 16;41(1):141–9. doi: 10.1007/s10545-017-0095-6 (PMC5786653; doi:10.1007/s10545-017-0095-6)
Supplement: Supplementary file 1 — (PDF 70 kb) [file 10545_2017_95_MOESM1_ESM.pdf]

Supplemental A

Phenotypic classification (Adapted from Arends et al (2016) with permission)

Patients were classified as classical or non-classical FD on the basis of their enzyme activity (men only) and the presence or absence of characteristic symptoms (Smid et al 2014). Men were considered to have a classical phenotype when they met the following criteria: 1) a GLA mutation, 2) enzyme activity ≤5% of the mean reference range, 3) ≥1 characteristic FD symptoms (i.e. Fabry neuropathic pain, angiokeratoma and/or cornea verticillata, for definitions see (van der Tol et al 2014)). Men not fulfilling these criteria were categorized as non-classical FD.

Women with a GLA mutation and ≥1 characteristic FD symptoms (i.e. Fabry neuropathic pain, angiokeratoma and/or cornea verticillata (van der Tol et al 2014)) were classified as having a classical phenotype. Women without these characteristic FD symptoms were classified as non-classical FD.

Classification on the basis of phenotypic features and residual enzyme activity was challenging in two groups of patients. It was decided that in these cases a final judgement was made by the treating physician. These groups were:

1) Patients with the N215S mutation: this group is especially prevalent in the UK. According to literature and physician experience, patients exhibit a non-classical (mostly cardiac) phenotype, but exceptions may occur. In this group of 90 patients, 12 had a characteristic symptom, but without confirmatory deficiency of GLA activity in leucocytes in men ($n = 5$). Notably, one of the N215S patients presented with severe renal disease at young age and had a renal transplantation at age 29. According to the judgement of the treating physician this patient was classified as classical FD while the other N215S patients were all classified as non-classical FD. Similarly, three patients with characteristic symptoms and the P389A mutation (1 man, 1 woman) or R112H (1 woman) mutation were discussed with the treating physician. These patients all had a late onset presentation, only minimal cornea verticillata (no other characteristic FD symptoms) and a family history of non-classical FD. Consequently they were classified as non-classical FD.

2) Men with slightly higher than 5% enzyme activity in the presence of 1 or more characteristic symptoms ($n = 13$). Residual enzyme activity ranged from 6% to 10% in leucocytes ($n = 10$), and from 6% to 20% in plasma ($n = 3$). All had at least one characteristic FD symptom and the majority had a relative with classical FD and consequently were considered having classical FD. In four men the enzyme activity and/or the data on characteristic FD symptoms were missing. These patients were classified as classical FD according to the opinion of the treating physician, which was mainly based on their family history.

Furthermore, we included three patients (one man, two women, all from the same family) with the A143T mutation. They were classified as having classical FD based on the

combination of characteristic deposits on renal biopsy or post mortem biopsy, the presence of one or more characteristic FD symptoms, low enzyme activity (3.9%, 21% and 38% respectively) and high plasma lysoGb3 concentrations (men: 35-50 nmol/l while receiving ERT; woman 1: 16 nmol/l while receiving ERT; woman 2: 8 nmol/l while not receiving ERT). In these cases, a combination of the A143T mutation and an unknown mutation and/or other (genetic) disease modifiers may have caused the classical FD presentation.

Supplemental table A

Criteria for phenotypic classification

Classical FD	
Men	Women
▪ A mutation in the GLA gene*<br>▪ ≥ 1 of the following characteristic Fabry disease symptoms: Fabry neuropathic pain, angiokeratoma and/or cornea verticillata<br>▪ Severely decreased or absent leukocyte AGAL activity (<5% of the normal mean)	▪ A mutation in the GLA gene<br>▪ ≥ 1 of the following characteristic Fabry disease symptoms: Fabry neuropathic pain, angiokeratoma and/or cornea verticillata
Non-classical FD	
▪ A mutation in the GLA gene, and not fulfilling the criteria for classical FD	

**The following genetic variants were not considered FD (neutral variants): A143T, P60L, D313Y, R118C, T385A, IVS0-10 C>T, the complex haplotype: IVS0-10 C>T/IVS4-16A>G/IVS6-22C>T. In patients in whom classification on the basis of these criteria was not feasible, the final judgement was made by the treating physician.*
