# Supplementary material for: Phenotype, disease severity and pain are major determinants of quality of life in Fabry disease: results from a large multicenter cohort study
Source: J Inherit Metab Dis. 2017 Oct 16;41(1):141–9. doi: 10.1007/s10545-017-0095-6 (PMC5786653; doi:10.1007/s10545-017-0095-6)
Supplement: Supplementary file 2 — (PDF 99 kb) [file 10545_2017_95_MOESM2_ESM.pdf]

Supplemental table B Health profile of first EQ-5D measurement per disease state

Disease state	No organ involvement	Neuropathic pain	Organ involvement	End stage renal disease	Cerebrovascular accident	Cardiac complication(s)	Multiple complications
Patients[#], n	31	21	221	7	16	45	18
Mobility	(Number of patients (%))						
1*	28 (90.3)	17 (81)	169 (76.5)	4 (57.1)	9 (56.2)	24 (53.3)	5 (27.8)
2	3 (9.7)	4 (19)	52 (23.5)	3 (42.9)	7 (43.8)	21 (46.7)	12 (66.7)
3	0 (0.0)	0 (0.0)	0 (0.0)	0 (0.0)	0 (0.0)	0 (0.0)	1 (5.6)
Self-care	(Number of patients (%))						
1	30 (96.8)	20 (95.2)	209 (94.6)	5 (71.4)	13 (81.2)	40 (88.9)	12 (66.7)
2	1 (3.2)	1 (4.8)	10 (4.5)	2 (28.6)	3 (18.8)	5 (11.1)	5 (27.8)
3	0 (0.0)	0 (0.0)	2 (0.9)	0 (0.0)	0 (0.0)	0 (0.0)	1 (5.6)
Usual activities	(Number of patients (%))						
1	25 (80.6)	11 (52.4)	143 (64.7)	4 (57.1)	7 (43.8)	20 (44.4)	5 (27.8)
2	5 (16.1)	10 (47.6)	69 (31.2)	3 (42.9)	9 (56.2)	24 (53.3)	11 (61.1)
3	1 (3.2)	0 (0.0)	9 (4.1)	0 (0.0)	0 (0.0)	1 (2.2)	2 (11.1)
Pain/discomfort	(Number of patients (%))						
1	20 (64.5)	8 (38.1)	108 (48.9)	5 (71.4)	5 (31.2)	19 (42.2)	2 (11.1)
2	9 (29.0)	8 (38.1)	96 (43.3)	2 (28.6)	10 (62.5)	22 (48.9)	15 (83.3)
3	2 (6.5)	5 (23.8)	17 (7.7)	0 (0.0)	1 (6.2)	4 (8.9)	1 (5.6)
Anxiety/Depression	(Number of patients (%))						
1	20 (64.5)	13 (61.9)	149 (67.4)	5 (71.4)	12 (75.0)	27 (60.0)	11 (61.1)
2	10 (32.2)	8 (38.1)	69 (31.2)	2 (28.6)	4 (25.0)	15 (33.3)	6 (33.3)
3	1 (3.2)	0 (0.0)	3 (1.4)	0 (0.0)	0 (0.0)	3 (6.7)	1 (5.60)

[#] Patients may have more than one EQ-5D per disease state and may contribute to more than one disease state

** 1 = No problems, 2 = Some/Moderate problems, 3 = Extreme problems*
