# Supplementary material for: Phenotype, disease severity and pain are major determinants of quality of life in Fabry disease: results from a large multicenter cohort study
Source: J Inherit Metab Dis. 2017 Oct 16;41(1):141–9. doi: 10.1007/s10545-017-0095-6 (PMC5786653; doi:10.1007/s10545-017-0095-6)
Supplement: Supplementary file 3 — (PDF 154 kb) [file 10545_2017_95_MOESM3_ESM.pdf]

Supplemental table C Comparison of health profile first EQ-5D of present study with literature

	Present study	Present study	Miners et al (2002)	Kind et al (1998)	Lamers et al (2006)
	FD (all patients)	FD (men with classical disease)	FD (pre-ERT cohort)	Sample general population	Sample general population
N	286	76	38	3395	298
Age, years (±SD)	42.5 (±15.5)	37.4 (±12.5)	37.2 (±9.2)	Unknown	43.4 (±15.0)
Men, n (%)	114 (39.8)	76 (100)	38 (100)	1562 (46)	152 (51.0)
Classical phenotype, n (%)	172 (60.1)	76 (100)	Unknown	-	-
Country	UK/NL	UK/NL	UK	UK	NL
Mobility	(Number of patients (%))				
1*	207 (72.4)	48 (63.2)	19 (50.0)	2424 (71.6)	258 (86.5)
2	78 (27.3)	27 (35.5)	18 (47.4)	620 (18.3)	40 (13.5)#
3	1 (0.3)	1 (1.3)	1 (2.6)	3 (0.1)	
Self-care	(Number of patients (%))				
1	261 (91.3)	64 (84.2)	28 (73.7)	3285 (95.8)	292 (98.0)
2	22 (7.7)	10 (13.2)	9 (23.7)	139 (4.1)	6 (2.0)#
3	3 (1.0)	2 (2.6)	1 (2.6)	5 (0.1)	
Usual activities	(Number of patients (%))				
1	173 (60.5)	40 (52.6)	17 (44.7)	2829 (83.7)	257 (86.2)
2	101 (35.3)	32 (42.1)	20 (52.7)	481 (14.2)	41 (13.8)#
3	12 (4.2)	4 (5.3)	1 (2.6)	70 (2.1)	
Pain/discomfort	(Number of patients (%))				
1	138 (48.3)	29 (38.2)	10 (26.3)	2268 (67.0)	193 (64.8)
2	127 (44.4)	43 (56.6)	21 (55.3)	988 (29.2)	105 (35.2)#
3	21 (7.3)	4 (5.3)	7 (18.4)	129 (3.8)	
Anxiety/depression	(Number of patients (%))				
1	191 (66.8)	51 (67.1)	19 (50.0)	2687 (79.1)	255 (85.6)
2	90 (31.5)	25 (32.9)	14 (36.8)	648 (19.1)	43 (14.4)#
3	5 (1.7)	0 (0.0)	5 (13.2)	62 (1.8)	

** 1 = No problems, 2 = Some/Moderate problems, 3 = Extreme problems*

N = Number of respondents, UK = United Kingdom, NL = Netherlands.

# Dutch sample of general population only provided combination of some/moderate and extreme problems as "any problems".
